# Coexistence of superconductivity and ferromagnetism in Sr_0.5_Ce_0.5_FBiS_2-*x*_Se_*x*_ (*x *= 0.5 and 1.0), a non-U material with *T*_*c*_ < T_FM_

**DOI:** 10.1038/srep37527

**Published:** 2016-11-28

**Authors:** Gohil S. Thakur, G. Fuchs, K. Nenkov, Zeba Haque, L. C. Gupta, A. K. Ganguli

**Affiliations:** 1 Department of Chemistry, Indian Institute of Technology, New Delhi, 110016, India; 2 Leibniz-Institut für Festkörper- und Werkstoffforschung Dresden, 01069, Germany; 3 Institute of Nano Science and Technology, Mohali, Punjab, 160064, India

## Abstract

We have carried out detailed magnetic and transport studies of the new Sr_0.5_Ce_0.5_FBiS_2-*x*_Se_*x*_ (0.0 ≤ *x* ≤ 1.0) superconductors derived by doping Se in Sr_0.5_Ce_0.5_FBiS_2_. Se–doping produces several effects: it suppresses semiconducting–like behavior observed in the undoped Sr_0.5_Ce_0.5_FBiS_2_, the ferromagnetic ordering temperature, *T*_FM_, decreases considerably from 7.5 K (in Sr_0.5_Ce_0.5_FBiS_2_) to 3.5 K and the superconducting transition temperature, *T*_*c*_, gets enhanced slightly to 2.9–3.3 K. Thus in these Se–doped materials, *T*_FM_ is marginally higher than *T*_*c*_. Magnetization studies provide evidence of bulk superconductivity in Sr_0.5_Ce_0.5_FBiS_2-*x*_Se_*x*_ at *x* ≥ 0.5 in contrast to the undoped Sr_0.5_Ce_0.5_FBiS_2_ (*x* = 0) where magnetization measurements indicate a small superconducting volume fraction. Quite remarkably, as compared with the effective paramagnetic Ce–moment (~2.2 *μ*_B_), the ferromagnetically ordered Ce–moment in the superconducting state is rather small (~0.1 *μ*_B_) suggesting itinerant ferromagnetism. To the best of our knowledge, Sr_0.5_Ce_0.5_FBiS_2-*x*_ Se_*x*_ (_*x*_ = 0.5 and 1.0) are distinctive Ce–based bulk superconducting itinerant ferromagnetic materials with ***T***_***c***_ < **T**_**FM**_. Furthermore, a novel feature of these materials is that they exhibit a dual and quite unusual hysteresis loop corresponding to both the ferromagnetism and the coexisting bulk superconductivity.

Traditionally, superconductivity and long–range ferromagnetism had been considered mutually exclusive (BCS pair–breaking and Meissner effect). For example, in several ternary materials RMo_6_X_8_ (X = S, Se)^1^ and RRh_4_B_4_^2^,^3^, studied in the early eighties, superconductivity was observed coexisting with long range antiferromagnetic order. But uniform ferromagnetism suppressed superconductivity at low temperatures^4^,^5^,^6^. The situation has changed strikingly over the last several years with the discovery of a number of materials that do exhibit coexistence of superconductivity and long-range ferromagnetism. In materials such as ErNi_2_B_2_C^7^,^8^ and RuSr_2_GdCu_2_O_8_^9^, localized 4*f*–moments (Er, Gd) are responsible for long-range ferromagnetism whereas 3*d*–conduction electrons carry superconductivity. In certain U-containing materials, such as UGe_2_^10^, URhGe^11^, UIr^12^, UCoGe^13^, the situation is drastically different. Here U–5*f* itinerant electrons are responsible for both superconductivity and ferromagnetism. These materials, with **T**_**FM**_ > ***T***_***SC***_, present an unusual and surprising scenario of coexistence, namely, superconductivity setting in an already ferromagnetically ordered host. In such cases, spin-triplet pairing (*p*-wave superconductivity) has been suggested (U-compounds such as UCoGe, URhGe and UGe_2_ have been proposed/considered *p*-wave ferromagnetic superconductors)^14^ to be compatible with itinerant ferromagnetism. For *p*-wave pairing, one needs to go beyond electron-phonon interaction (pairing mechanism in conventional superconductivity) with Cooper-pairing mediated via spin fluctuations. The material UCoGe is of particular interest from the viewpoint of the present work. In this material the paramagnetic effective moment of U is ~1.7 *μ*_B_ whereas the ferromagnetic ordered moment of U is drastically reduced, 0.03 *μ*_B_^13^. The materials under investigation (the title compounds) in this work, exhibit coexisting superconductivity and itinerant ferromagnetic properties, as we shall see below, similar to those of UCoGe.

The parent material Sr_0.5_Ce_0.5_FBiS_2_ of the title compounds Sr_0.5_Ce_0.5_FBiS_2-*x*_Se_*x*_ belongs to a small class AFBiS_2_ (A = Sr and Eu)^15^,^16^ of a larger family of BiS_2_ layered tetragonal materials LnOBiS_2_^17^,^18^ (*P*4/*nmm*) recently shown to exhibit superconductivity^19^,^20^,^21^,^22^,^23^,^24^,^25^. SrFBiS_2_ is derived by replacing Ln–O layers by Sr–F layers. Its structure essentially consists of alternate stacking of conducting BiS_2_ layers and blocking (insulating) layer LnO/SrF^15^,^17^,^19^. Electron carriers are doped into the superconducting BiS_2_ layers employing the commonly used doping strategy, namely, replacing O partially by F, for instance LaO_0.5_F_0.5_BiS_2_ exhibits superconductivity at *T*_*c*_ ~ 2.8 K^19^. In AFBiS_2_ electron doping and eventual superconductivity is achieved/enhanced by Ln (La and Ce) doping at A sites^26^,^27^,^28^,^29^. Structurally, these materials are quite similar to high-*T*_*c*_ cuprates and iron pnictides and superconductivity is quite robust as evident from numerous studies on various site substitutions^24^,^30^,^31^,^32^. *T*_*c*_ is enhanced in LnO_1-*x*_F_*x*_BiS_2_ by chemical pressure via partial or complete substitution of La by a smaller rare-earth (Ln = Ce, Pr, Nd, Sm and Yb)^23^,^33^,^34^,^35^,^36^. YbO_0.5_F_0.5_BiS_2_ has the highest *T*_*c*_ = 5.4 K among LnO_1-*x*_F_*x*_BiS_2_ and, interestingly, it undergoes an antiferromagnetic transition (T_N_ ~ 2.7 K) also^23^. Se substitution has been realized in LaO_0.5_F_0.5_BiS_2-*x*_Se_*x*_^37^ where an enhancement of *T*_*c*_ is observed with maximum *T*_*c*_ of 3.8 K for LaO_0.5_F_0.5_BiSSe composition (*x* = 1.0). *T*_*c*_ decreases on further Se substitution. For other rare earths (Ce and Nd), however, the effect of Se on *T*_*c*_ is different. In Ce(O/F)BiS_2_ enhancement in *T*_*c*_ is only marginal (2.4 to 2.6 K)^38^. Se substitution induces bulk superconductivity in La and Ce materials. In Nd(O/F)BiS_2_ and Bi_4_O_4_S_3_, Se doping has been shown to depress *T*_*c*_^32^,^39^. Se substitution in AFBiS_2_ has not been tried so far. Under applied pressure, *T*_*c*_ is enhanced in LnO_1-*x*_F_*x*_BiS_2_ and A_1-*x*_Ln_*x*_FBiS_2_ (Ln = La, Ce, Pr and Nd; A = Sr and Eu) upto a maximum of 10 K^40^,^41^,^42^,^43^,^44^,^45^,^46^. The Bi-S_2_ materials are BCS-like and probably have *s*-wave pairing symmetry^47^,^48^,^49^,^50^,^51^. But there is yet no consensus on the origin of superconductivity in these materials^31^.

Very recently, ferromagnetism and superconductivity have been reported to coexist in CeO_1-*x*_ F_*x*_BiS_2_ and Sr_1-*x*_Ce_*x*_FBiS_2_ with *T*_*c*_ ~ 2.5–4 K and *T*_FM_ ~4–8 K^20^,^27^,^52^,^53^,^54^. As these materials have layered structure, magnetism originates in the Ce−O (or Sr/Ce−F) layers and conduction occurs in BiS_2_ layers. In Sr_0.5_Ce_0.5_FBiS_2_, the parent materials for our Se–added materials Sr_0.5_Ce_0.5_FBiS_2-*x*_Se_*x*_, Ce–substitution provides conduction electrons as well as gives rise to long range magnetic order^27^. Ferromagnetic order takes place at a higher temperature (7.5 K) and superconductivity sets in at a lower temperature (2.8 K) in an already ferromagnetically ordered lattice. We report here the effect of substitution of larger isovalent Se ion at the S site on the magnetic and superconducting properties of Sr_0.5_Ce_0.5_FBiS_2_. Se–doping leads to a modest enhancement of *T*_*c*_ (upto 3.3 K) and a significant depression of *T*_FM_ (down to 3.5 K). Thus Se–doping moves *T*_*c*_ and *T*_FM_ in opposite directions, bringing them in closer proximity in temperature. We believe the ferromagnetism in our materials is itinerant just as it is in UCoGe^13^, namely, high Ce-paramagnetic moment (~2.2 μ_B_) and small saturation Ce-magnetic moment (0.1 μ_B_). To the best of our knowledge, the materials Sr_0.5_Ce_0.5_FBiS_2-*x*_Se_*x*_, *x* = 0.5, 1.0 are unique Ce-containing materials exhibiting coexisting bulk superconductivity and itinerant ferromagnetism. Thus our observation of the coexistence of superconductivity and itinerant ferromagnetism in Sr_0.5_Ce_0.5_FBiS_2-*x*_Se_*x*_ is a timely discovery, in that it puts U- and Ce on equal footing in this respect also.

## Results and Discussion

### PXRD characterization

**Figure 1:**
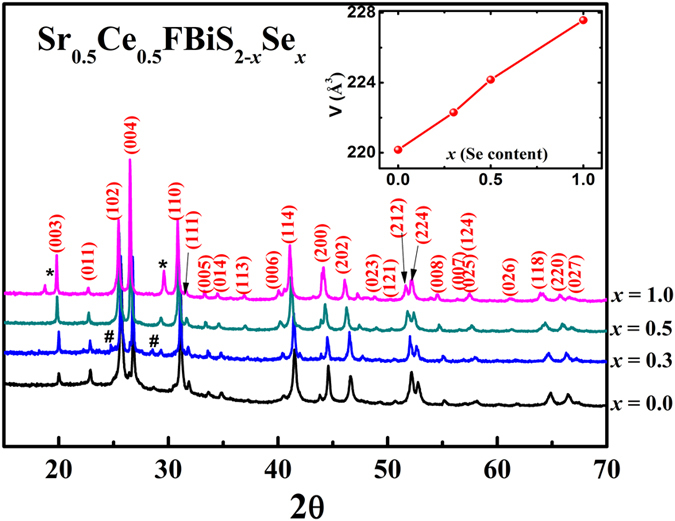
Powder X-ray diffraction of Sr_0.5_Ce_0.5_FBiS_2-*x*_Se_*x*_(*x* = 0.0, 0.3, 0.5 and 1.0). Symbols (*) and (**#**) indicate impurity phases Bi_2_Se_3_ and Bi_2_S_3_ respectively. Inset shows the variation of cell volume with Se content.

PXRD patterns of all the Sr_0.5_Ce_0.5_FBiS_2-*x*_Se_*x*_ (*x* = 0.0, 0.3, 0.5 and 1.0) compositions are shown in Fig. 1. All the peaks could be easily indexed on the basis of a SrFBiS_2_ type tetragonal unit cell (SG: *P*4/*nmm*). Minor peaks corresponding to the impurity of Bi_2_S_3_ (#) and Bi_2_Se_3_ (*) were also observed for composition with *x* > 0. The estimated impurity phase of Bi_2_S_3_ was ~4% observed in *x* = 0.3 composition whereas the amount of Bi_2_Se_3_ was ~6% and ~14% in *x* = 0.5 and *x* = 1.0 composition respectively. It is evident from X-ray studies that the impurities increase with the increase of Se content. The samples with *x* > 1.0 were obtained as multiphase products. This indicates a Se solubility limit of *x *~ 1.0. Lattice parameters *a* and *c* show an expected increase upon Se doping (*a* = 4.0886(2) Å, *c* = 13.4143(8) Å for *x* = 0.5 and *a* = 4.1057(1) Å, *c* = 13.4756(8) Å for *x* = 1.0) resulting in the monotonous unit cell expansion (inset in Fig. 1). Compositional analysis on *x* = 0.5 and 1.0 samples gives a stoichiometry close to the nominal value for both the compositions (Figure S1 in supplementary material (SM)). For *x* = 1.0 sample, the Se:S ratio was slightly less than 1, possibly due to the formation of small amount of the impurity phase Bi_2_Se_3_, which is non-magnetic and insulating under ambient pressure^55^. It does not interfere with superconducting and magnetic properties of the materials under investigation.

### Resistivity

**Figure 2:**
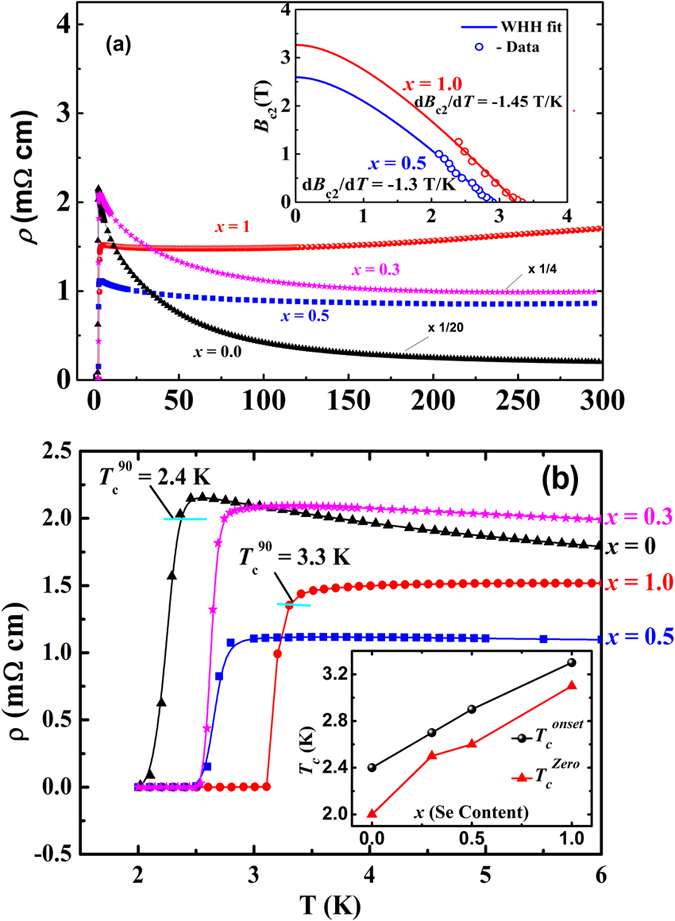
Variable temperature resistivity curves for Sr_0.5_Ce_0.5_FBiS_2-*x*_Se_*x*_; *x* = 0, 0.3, 0.5 and 1.0. The data for *x* = 0 and 0.3 have been divided by 20 and 4 respectively (**a**) in temperature range 2–300 K and (**b**) in low temperature range. Inset of (**a**) shows the upper critical field (*B*_c2_) versus temperature (T) curve for the *x* = 0.5 and 1.0 compositions (open circles) along with the WHH fit (solid lines). Inset of (**b**) shows the variation of *T*_*c*_^onset^ and *T*_*c*_ (ρ = zero) as a function of Se-doping.

Resistivity of the materials as a function of temperature is shown in Fig. 2. In the normal state, resistivity of Sr_0.5_Ce_0.5_FBiS_2-*x*_Se_*x*_ with *x* = 0 and 0.3 exhibit semiconducting–like temperature dependence, namely, it increases with the decrease of temperature just before the onset of superconducting transition at 2.4 K and 2.7 K respectively as shown in Fig. 2(a). Note that the resistivity values for *x* = 0 and 0.3 were divided by a factor of 20 and 4 respectively for the purpose of clarity. In the higher Se–doped materials, *x* = 0.5 and *x* = 1.0, this semiconducting behavior is progressively subdued and metallic conductivity is observed in the normal state. Superconductivity sets in at *T*_*c*_ = 2.9 and 3.3 K in materials with *x* = 0.5 and 1.0 respectively. Our estimate of *T*_*c*_^onset^ is based on a 90% criterion as shown in Fig. 2(b). Se–doping clearly *enhances T*_*c*_ by ~1 K (inset of Fig. 2(b)). In the material with *x* = 1.0, a sharp superconducting transition is observed with a transition width Δ*T* = 0.2 K. Similar small enhancement in *T*_*c*_ with Se substitution was previously observed in LnO_1*-x*_F_*x*_BiS_2_ (Ln = La and Ce)^38^,^56^,^57^. This enhancement in *T*_*c*_ is attributed to the in-plane chemical pressure induced by the Se substitution at S sites as elucidated by Mizuguchi *et al*.^58^. The plot of upper critical field, *B*_*c*2_ (T) as a function of temperature is given in the inset of Fig. 2(a). We estimated *B*_*c2*_ below 2 K using a standard single-band Werthamer–Helfand–Hohenberg (WHH) formula with the Maki parameter^59^ *α* = 0. Upper critical field, *B*_*c*2_(0) at *T* = 0 is estimated to be 2.6 T for *x* = 0.5 and 3.3 T for *x* = 1.0. These *B*_*c*2_ values are atleast twice higher than those reported for the Se–free samples Sr_0.5_Ln_0.5_FBiS_2_^26^,^27^. Enhancement of *T*_*c*_ and *B*_*c*2_ in the Se–doped samples clearly indicates that Se atoms have entered the lattice. Enhancement of *B*_*c*2_ implies reduction of the coherence length or stronger impurity scattering due to Se doping in Sr_0.5_Ce_0.5_FBiS_2_.

### Magnetic susceptibility in low field of 10 Oe

**Figure 3:**
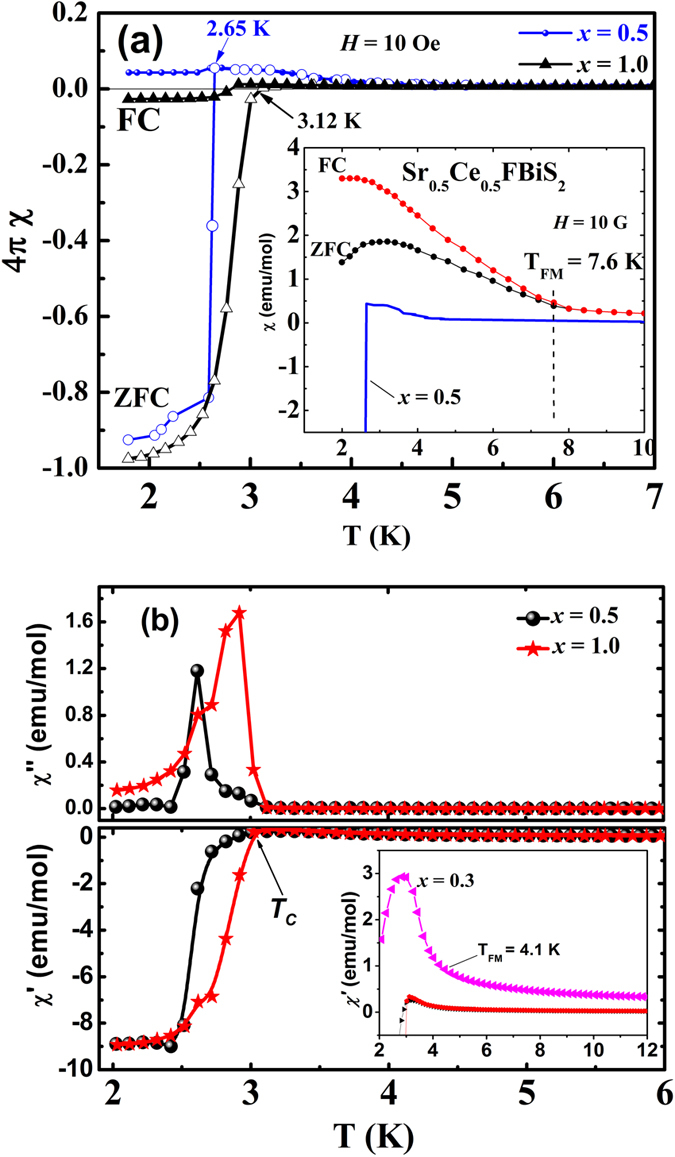
(**a**) Variable temperature *dc* susceptibility in ZFC and FC protocols for Sr_0.5_Ce_0.5_FBiS_2-*x*_Se_*x*_ (*x* = 0.5, 1.0) in an applied field of 10 Oe. Inset shows *dc* susceptibility for *x* = 0.0 sample in comparison with *x* = 0.5 sample in the same units (emu/mol) and (**b**) Real and imaginary parts of *ac* susceptibility for *x* = 0.5 and 1.0 samples. Inset shows *ac* susceptibility for *x* = 0.3 sample in comparison with *x* = 0.5 and 1.0 samples.

Figure 3(a) shows dc susceptibility of Sr_0.5_Ce_0.5_FBiS_2-x_Se_x_ (*x* = 0.5 and 1.0), in both the field-cooled (FC) and the zero field-cooled (ZFC) conditions in an applied field of 10 Oe. Clear diamagnetic signal, of magnitude close to the theoretical value, for both the *x* = 0.5 and 1.0 compositions is observed in ZFC condition (Fig. 3a) establishing the superconducting state. Poor Meissner response in both cases is possibly due to flux pinning. A superconducting volume fraction of >95% is estimated for both *x* = 0.5 and 1.0 compositions. In several studies^60^,^61^,^62^,^63^,^64^,^65^ on a variety of materials, such large diamagnetic superconducting signals have been observed and have been considered suggesting bulk superconductivity therein. Inset of Fig. 3(a) shows dc susceptibility of the Se free sample Sr_0.5_Ce_0.5_FBiS_2_ (*x* = 0.0) which shows a ferromagnetic behavior with Curie temperature ~7.5 K, similar to that reported earlier by Li *et al*.^27^. A weak drop in the ZFC susceptibility below 3 K is due to the superconducting transition that was also observed in our resistivity measurements. Such a weak diamagnetic signal rules out bulk superconductivity in parent sample *x* = 0.0 and is consistent with weak superconductivity. Figure 3(b) shows both the real and the imaginary parts of the ac susceptibility. A large superconducting screening indicates bulk superconductivity. Moreover a larger imaginary part of the signal indicates a considerable energy loss due to movement of vortices. Such a behavior cannot be explained if superconductivity is present only in thin surface layers. As deduced from these measurements, superconducting transition temperature increases from *T*_*c*_^onset^ = 2.65 K for *x* = 0.5 to *T*_*c*_^onset^ = 3.20 K for *x* = 1 which corroborates well with the resistivity data described above. It must be pointed out that in the earlier measurements on Se–free Sr_0.5_Ce_0.5_FBiS_2_^27^,^44^ materials diamagnetic signal was not observed and the occurrence of superconductivity was inferred from the resistivity measurements only. Inset of Fig. 3b shows the low temperature *ac* susceptibility (real part) data for the compositions with *x* = 0.3 in comparison with *x* = 0.5 and 1.0 samples. It shows a ferromagnetic behavior similar to *x* = 0.0 with a reduced Curie temperature T_FM_ = 4.1 K. No diamagnetic signal was observed indicating that similar to the parent compound *x* = 0.3 is also a weak superconductor. A clear diamagnetic signal is observed only for *x* = 0.5 and 1.0 samples.

Further, in Fig. 3(a), a weak magnetic anomaly is discernible at 3.5 K for the sample *x* = 0.5 which corresponds to a ferromagnetic transition as evidenced in our high field measurements, (see below), for both the samples *x* = 0.5 and *x* = 1.0. This anomaly is not observed clearly for the sample *x* = 1.0. *T*_*c*_ and *T*_*FM*_ were ascertained from the derivative plots of susceptibility (see Figure S2 in SM). It is evident from the susceptibility studies that Se substitution depresses ferromagnetic ordering and enhances *T*_*c*_ in Sr_0.5_Ce_0.5_FBiS_2-*x*_Se_*x*_.

### High field DC magnetization measurements

Magnetic susceptibility *χ*(*T*), measured in an applied field of 10 kOe, and its inverse in Sr_0.5_Ce_0.5_FBiS_2-*x*_Se_*x*_ (*x* = 0.5 and 1.0) is presented in the Figure S3 in SM. By fitting the data above 50 K to the Curie–Weiss law *χ*(*T*) = *χ*_o_ + C/(*T*−θ), the paramagnetic effective magnetic moments obtained for the two samples are: *μ*_eff_ = 2.22 *μ*_B_ for *x* = 0.5 and 2.29*μ*_B_ for *x* = 1.0 (see Figure S3 in SM). These values are close to the theoretical value 2.54 *μ*_B_ for free Ce^3+^ ions. Thus Ce–ions are in trivalent (or nearly trivalent state) state.

**Figure 4:**
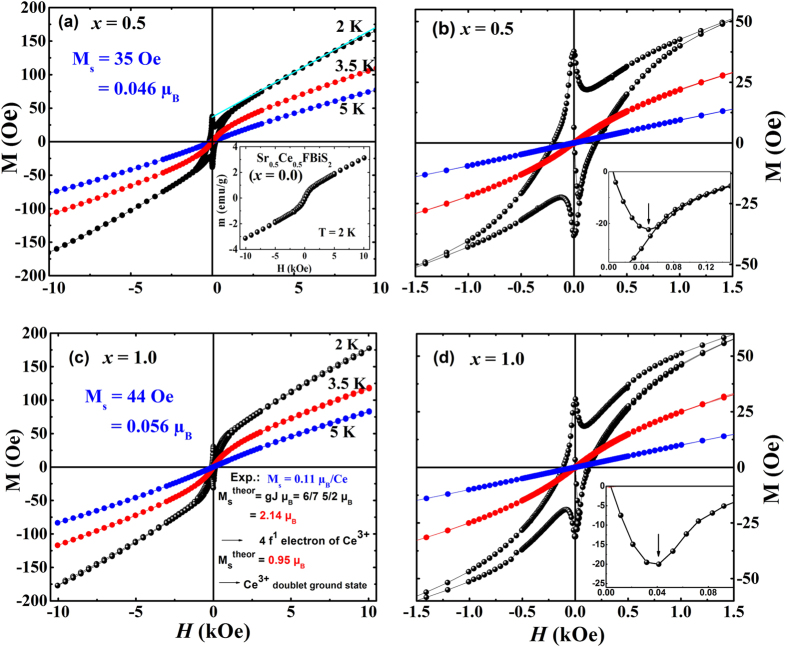
Hysteresis loops at different temperatures for Sr_0.5_Ce_0.5_FBiS_1.5_Se_0.5_ and Sr_0.5_Ce_0.5_FBiSSe in *H* ≤ 10 kOe (**a**) and (**c**) and *H* ≤ 1.5 kOe (**b**) and (**d**). The superconducting loop is superimposed on the ferromagnetic loop at 2 K. Inset in Fig. 4(a) shows the ferromagnetic hysteresis loop for *x* = 0 composition. Insets in Fig. 4(b) and (d) show initial diamagnetic signal with arrows indicating lower critical field, *H*_*c1*_.

We display in Fig. 4 the results of our magnetization measurements, at a few selected temperatures 5 K, 3.5 K and 2 K, in Sr_0.5_Ce_0.5_FBiS_1.5_Se_0.5_ and Sr_0.5_Ce_0.5_FBiSSe. At 5 K, magnetization M varies linearly with applied magnetic field, suggesting a paramagnetic state (no magnetic order). At 3.5 K, *M* is no longer linear in *H* in the low field region and shows a sign of a ferromagnetic behavior. Ferromagnetic state is clearly observed at a lower temperature 2 K and, remarkably, at this temperature in both the samples, we observe a ferromagnetic hysteresis loop and a superimposed superconducting hysteresis loop, demonstrating unambiguously the coexistence of ferromagnetism and bulk superconductivity. A dual loop, displaying the two ordered states, superconductivity and ferromagnetism, with such clarity, is a novel feature of this material. In UCoGe, ferromagnetic hysteresis is observed in the ferromagnetic state (*T*_*c*_ < *T* < *T*_FM_) but no superconducting hysteresis loop as such was observed^66^. Inset of Fig. 4a shows the isothermal magnetization (at 2 K) for *x* = 0.0 where only a ferromagnetic hysteresis loop is observed. In the inset of Fig. 4(b) and (d) the diamagnetic response is clearly seen in the virgin low field region (from which *H*_*c1*_ is easily estimated to be ~44 Oe and 40 Oe for *x* = 0.5 and 1.0 respectively. It is important to point out that in the selenium-free compound Sr_0.5_Ce_0.5_FBiS_2_ (*T*_c_ ~ 2.6 K &*T*_FM_ ~ 7.5 K)^27^,^44^ and in a similar material Ce(O, F)BiS_2_ (*T*_c_ ~ 2.5–4 K & T_FM_ ~ 6.5–7.5 K)^20^,^52^,^53^,^67^ no superconducting hysteresis loop was observed. This is consistent with our own results on Sr_0.5_Ce_0.5_FBiS_2_ (inset of Fig. 4a). Thus Se–doping has created crucial changes in the superconducting and magnetic properties of the parent material Sr_0.5_Ce_0.5_FBiS_2_. Observation of superconducting loop is a good indication of bulk superconductivity.

Dual hysteresis loop has been observed very recently in [(Li_1-*x*_Fe_*x*_)OH](Fe_1-*y*_Li_*y*_)Se^68^. However, there is a fundamental difference in this material and our samples, namely, in this case, *T*_*c*_ (~43 K) >>*T*_FM_ (10 K) whereas in our case *T*_*c*_ < *T*_FM_ and hence, superconductivity sets in an already ferromagnetically ordered lattice. Further, in our case, superconductivity appears just at the border of ferromagnetic transition (*T*_*FM*_ is only marginally higher than *T*_*c*_) whereas in the above–mentioned material, superconductivity and ferromagnetism are far separated in temperature. In CeFeAs_1-*x*_P_*x*_O_0.95_F_0.05_, coexistence of superconductivity and ferromagnetism (with *T*_*c*_ > *T*_FM_) has been observed^69^ in a limited doping range. In this case, however, Ce carries almost full moment and the system is not an itinerant ferromagnet.

The spontaneous magnetization *M*_s_ is estimated by linear extrapolation of the high–field data to *H* = 0 (Fig. 4(a) and (c)). From the estimated *M*_s_, we obtain at *T* = 2 K, the spontaneous Ce–moment *μ*_0_ ~ 0.09 *μ*_B_ for the sample *x* = 0.5 and 0.11 *μ*_B_ for the sample *x* = 1.0. These values are quite small as compared with what is expected for free Ce^3+^ ion. We may note here that in Ce(O, F)BiS_2_ a reduced moment *M*_s_ = 0.52 *μ*_B_/Ce was reported^53^ which, possibly, suggests that in this case Ce–ions may be in the crystal–field split doublet state (localized moment). In our case, we observe a drastically reduced, but non–zero, Ce–moment.

The transition of the high Ce–paramagnetic effective moment *μ*_eff_ ~ 2.2 *μ*_B_ to a small ordered moment *μ*_0_ ~ 0.1 *μ*_B_ in the superconducting state is an important observation as the drastic loss of Ce–moment signals a delocalization of the 4*f* electrons concurrent with the appearance of superconductivity. Thus, 4*f*–electrons may also be involved in superconductivity in these materials. A *high* ratio *μ*_eff_/*μ*_0_ (~22) implies an itinerant ferromagnetic state^13^,^70^ in both materials Sr_0.5_Ce_0.5_FBiS_2-*x*_Se_*x*_, *x* = 0.5 and *x* = 1.0. Further, as Ce–atoms are responsible both for ferromagnetism and coexisting bulk superconductivity we think the two phenomena can coexist uniformly. These materials fill the glaring void, namely, so far no Ce–based material has been hitherto known exhibiting superconductivity within the itinerant ferromagnetic state.

### Specific heat

**Figure 5:**
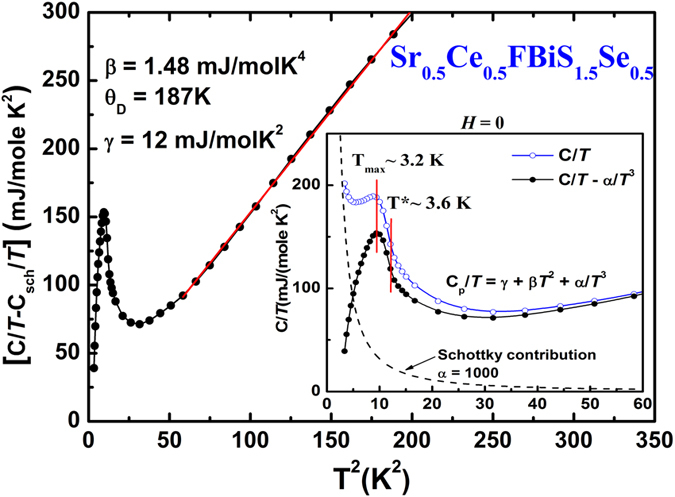
Temperature dependence of Schottky corrected specific heat C/*T* vs.*T*^2^ for *x* = 0.5 sample at *H* = 0. Red line is the linear fit to the equation C/T = *γ* + *β* T^2^. Inset shows C/*T* data before (blue circle) and after subtraction (black circle) of a Schottky contribution which is represented by the dashed line.

Figure 5 shows the temperature dependence of specific heat of Sr_0.5_Ce_0.5_FBiS_1.5_Se_0.5_ (*x* = 0.5) in the low temperature range 2–16 K. Inset shows C/*T* data before (blue circle) and after subtraction (black circle) of a Schottky contribution which was approximated by the dashed line. A broad peak, not *λ*–shaped as expected for a ferromagnet, centered at 3.2 K (inset of Fig. 5) is observed from which, following Li *et al*.^27^, ferromagnetic ordering temperature *T*_FM_ ~ 3.6 K is obtained. As *T*_FM_ and *T*_*c*_ are quite close, separate anomalies of the two transitions, the magnetic and the superconducting, are not resolved. It should be pointed that in similar systems like CeO_0.5_F_0.5_BiS_2_ and YbO_0.5_F_0.5_BiS_2_ specific heat anomaly around *T*_*c*_ is not observed^23^ and the anomaly observed in Sr_0.5_Ce_0.5_FBiS_2_ is extremely small^53^. Red line in the main panel of Fig. 5 obeys the equation: C/*T* = *γ* + *βT *^2^ yielding the Debye temperature θ_D_ = 187 K and the Sommerfeld coefficient *γ* = 12 mJ/(K^2^mol Ce). This *γ* value of Sr_0.5_Ce_0.5_FBiS_1.5_Se_0.5_ is much smaller than that of Sr_0.5_Ce_0.5_FBiS_2_ (*γ* = 117 mJ/(K^2^mol Ce))^27^. However, it is larger by a factor of 5–10 as compared to Sr_1-*x*_La_*x*_FBiS_2_ (*γ* < 2 mJ/mol-K^2^)^26^,^71^,^72^ and La_1−*x*_M_*x*_OBiS_2_ (M = Ti, Zr, Th) (*γ* ∼ 0.58–2.21 mJ/mol K^2^)^24^. Ce 4*f*–electrons are responsible for the increased density of states at the Fermi level in Sr_0.5_Ce_0.5_FBiS_1.5_Se_0.5_ as compared to that in other BiS_2_ system without Ce. Therefore, the higher *γ* value in the normal state of Sr_0.5_Ce_0.5_FBiS_2_ is attributed to the electronic correlation effect of Ce–4*f* electrons which are reduced in the Se–doped sample. From the specific heat measurements we get an entropy per Ce atom of only about 4% of the expected value for *J* = 5/2. The low magnetic entropy (*S*_*m*_ = 0.04*R*ln6) is consistent with weak itinerant ferromagnetism in Sr_0.5_Ce_0.5_FBiS_1.5_Se_0.5_. This situation is similar to that in UCoGe^13^.

**Figure 6:**
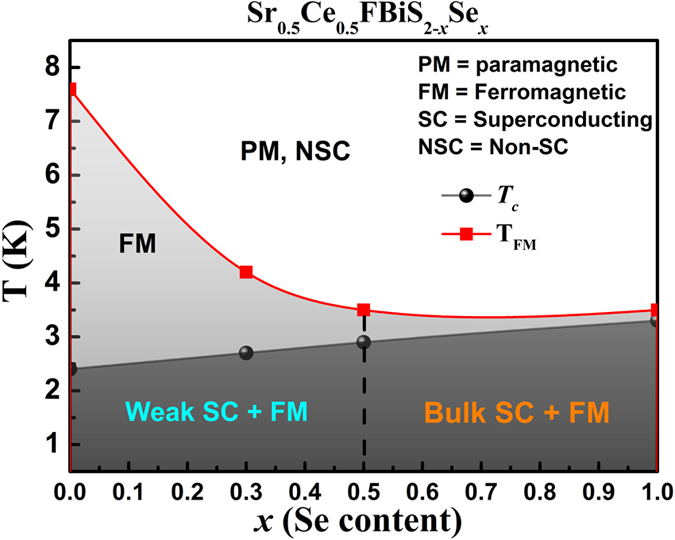
Ferromagnetism and superconductivity phase diagram of Sr_0.5_Ce_0.5_FBiS_2-*x*_Se_*x*_ (0 ≤ x ≤ 1).

With the help of our experimental data, we construct a ferromagnetism/superconductivity phase diagram of the system Sr_0.5_Ce_0.5_FBiS_2-*x*_Se_*x*_ (0 ≤ *x* ≤ 1) which is shown in Fig. 6. Upon doping Se ferromagnetic ordering temperature (T_FM_) decrease along with a concomitant increase in the *T*_*c*_. Ferromagnetism and weak superconductivity are observed for *x* < 0.5. In the two materials *x* = 0.5 and *x* = 1.0, bulk superconductivity is observed coexisting with ferromagnetism. The dual hysteresis loops, shown in Fig. 4, are observed for these samples. T_FM_ and *T*_*c*_lie in close proximity in the *x* = 1.0 composition.

After submission of this manuscript we came across a report on the coexistense of superconductivity and ferromagnetism in CsEuFe_4_As_4_ compound^73^. A dual hysteresis loop has been observed in this compound also. The superconducting loop, however, is not so prominent. This remark has been added in the revised version of the manuscript.

## Concluding Remarks

We have observed superconductivity (*T*_*c*_ ~ 3.0 K) and itinerant ferromagnetism (*T*_*FM*_ ~ 3.5 K) coexisting in the new materials Sr_0.5_Ce_0.5_FBiS_2-*x*_Se_*x*_ at *x* ≥ 0.5. Thus in these materials, superconductivity occurs much closer to the border of ferromagnetism than in UCoGe. A novel feature of these materials, as compared with the other ferromagnetic superconductors reported so far, is a dual hysteresis loop corresponding to both the coexisting bulk superconductivity and ferromagnetism. Thus Sr_0.5_Ce_0.5_FBiS_2-*x*_Se_*x*_ is an important and timely addition to the exciting Ce–based materials exhibiting coexisting superconductivity and magnetism. The materials Sr_0.5_Ce_0.5_FBiS_2-*x*_Se_*x*_ are potential candidates for the unconventional *p*–wave superconductivity^74^ and deserve to be further pursued in this regard. We are making efforts to grow single crystals of these materials. In single crystals (if we succeed to grow) or else in polycrystalline materials, we would carry out studies such as NMR, MuSR, neutron diffraction and Andreev reflection to throw further light on the coexistence of superconductivity and ferromagnetism and nature of the superconducting state (*p*-wave or *s*-wave) in these materials.

## Methods

Polycrystalline compounds of the series Sr_0.5_Ce_0.5_FBiS_2-*x*_Se_*x*_ (*x* = 0.0, 0.3, 0.5 and 1.0) were prepared by the usual solid state synthesis procedure as reported elsewhere^28^,^35^. SrF_2_, Bi and Se powder, pre-reacted Ce_2_S_3_ and Bi_2_S_3_ powder were thoroughly mixed, pelletized and sealed in quartz tube under vacuum. The tubes were then heated twice at 800 ^o^C for 24 hours with an intermediate grinding. The end products were black/dark grayish in color. Phase purity of all the compositions was checked by powder X–ray diffraction (PXRD) technique using Cu−K*α* radiation source. Temperature dependent resistivity, magnetization and specific heat measurements were performed using a 14 T PPMS (Quantum Design). The specific heat was measured using a relaxation technique.

## Additional Information

**How to cite this article**: Thakur, G. S. *et al*. Coexistence of superconductivity and ferromagnetism in Sr_0.5_Ce_0.5_FBiS_2-*x*_Se_*x*_ (*x* = 0.5 and 1.0), a non-U material with *T_c_* < T_FM_. *Sci. Rep.* **6**, 37527; doi: 10.1038/srep37527 (2016).

**Publisher’s note:** Springer Nature remains neutral with regard to jurisdictional claims in published maps and institutional affiliations.

## Supplementary Material

Supplementary Information
