# Supplementary material for: Coexistence of superconductivity and ferromagnetism in Sr0.5Ce0.5FBiS2-xSex (x = 0.5 and 1.0), a non-U material with Tc < TFM
Source: Sci Rep. 2016 Nov 28;6:37527. doi: 10.1038/srep37527 (PMC5124956; doi:10.1038/srep37527)

# Coexistence of superconductivity and ferromagnetism in $Sr_{0.5}Ce_{0.5}FBiS_{2-x}Se_x$ ($x$ = 0.5 and 1.0), the non-U material with $T_c < T_{FM}$

Gohil S. Thakur[1], G. Fuchs[2], K. Nenkov[2], Zeba Haque[1], L. C. Gupta[1‡], and A. K. Ganguli[1,3]*

[1]Department of Chemistry, Indian Institute of Technology, New Delhi, India, 110016

[2]Leibniz-Institut für Festkörper- und Werkstoffforschung Dresden, Germany, 01069

[3]Institute of Nano Science and Technology, Mohali, Punjab, India, 160064

**Figure S1.** Compositional analysis of $Sr_{0.5}Ce_{0.5}FBiS_{2-x}Se_x$ ($x$ = 0.5, 1.0) samples by SEM–EDAX. The typical spectra show the presence of all the constituent elements in the selected region (inset). Many such regions were selected and all the monitored regions had acceptable S and Se contents, thus eliminating the possibility of any phase separation.

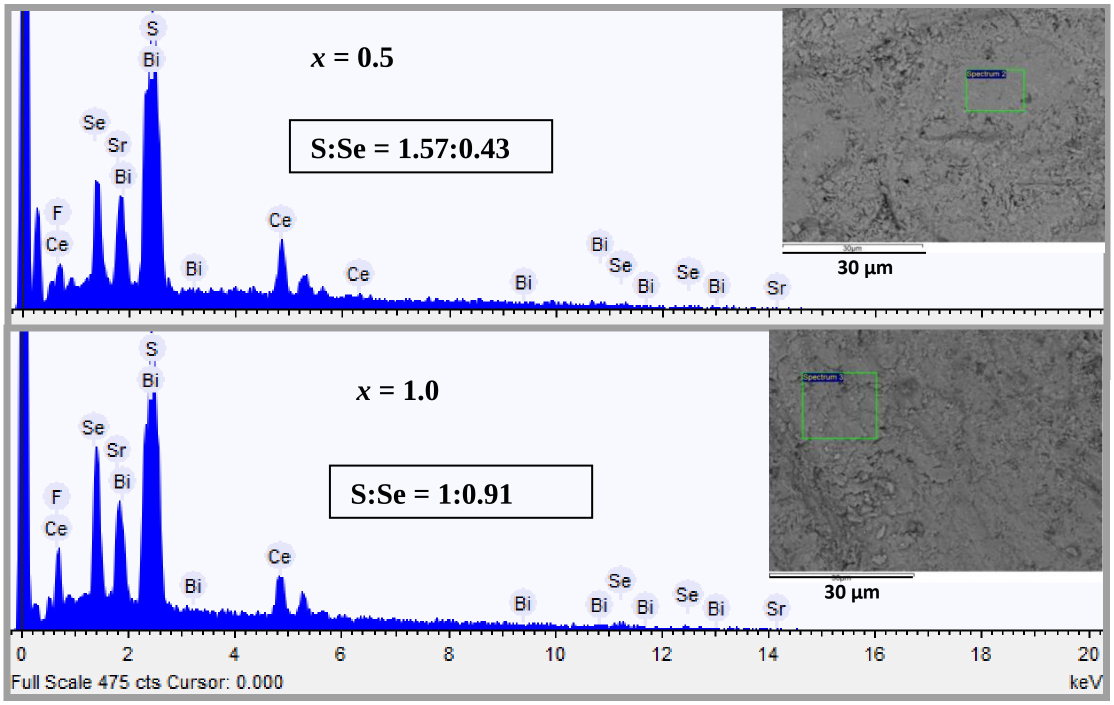

**Table S1. Elemental ratio taken at several regions for $Sr_{0.5}Ce_{0.5}FBiS_{1.5}Se_{0.5}$**

| Experiment # / Elements | 1 | 2 | 3 | 4 | 5 | 6 | Average |
|---|---|---|---|---|---|---|---|
| Fluorine | 13.11 | 20.32 | 18.06 | 22.72 | 21.34 | 24.45 | 20.00 |
| Sulfur | 33.16 | 35.01 | 28.47 | 29.06 | 29.59 | 32.16 | 31.24 |
| Selenium | 10.01 | 8.77 | 9.71 | 7.25 | 8.62 | 8.19 | 8.76 |
| Strontium | 9.45 | 9.01 | 7.07 | 10.74 | 10.15 | 10.06 | 9.41 |
| Cerium | 6.12 | 9.85 | 7.51 | 10.51 | 12.78 | 11.09 | 9.72 |
| Bismuth | 28.13 | 16.99 | 29.18 | 19.53 | 17.42 | 14.05 | 20.88 |

**Average composition: $Sr_{0.48}Ce_{0.49}FBi_{1.05}S_{1.57}Se_{0.43}$**

**Table S2. Elemental ratio taken at several regions for $Sr_{0.5}Ce_{0.5}FBiSSe$**

| Experiment # / Elements | 1 | 2 | 3 | 4 | 5 | 6 | Average |
|---|---|---|---|---|---|---|---|
| Fluorine | 13.62 | 21.32 | 17.06 | 25.72 | 19.34 | 23.05 | 20.02 |
| Sulfur | 22.16 | 23.01 | 19.47 | 14.06 | 20.59 | 21.16 | 20.07 |
| Selenium | 15.21 | 19.71 | 14.71 | 22.25 | 19.62 | 18.09 | 18.26 |
| Strontium | 8.45 | 10.11 | 10.07 | 8.74 | 11.15 | 9.06 | 9.59 |
| Cerium | 7.22 | 8.85 | 8.51 | 9.51 | 11.08 | 9.49 | 9.71 |
| Bismuth | 33.33 | 16.99 | 30.18 | 19.53 | 18.42 | 19.15 | 22.93 |

**Average composition: $Sr_{0.48}Ce_{0.46}F_{1.01}Bi_{1.14}S_{1.00}Se_{0.91}$**

**Figure S2.** Temperature dependent magnetic susceptibility and its derivative indicating the $T_c$ and $T_{FM}$ for $Sr_{0.5}Ce_{0.5}FBiS_{2-x}Se_x$ (a) $x$ = 0.5 and (b) $x$ = 0.1.

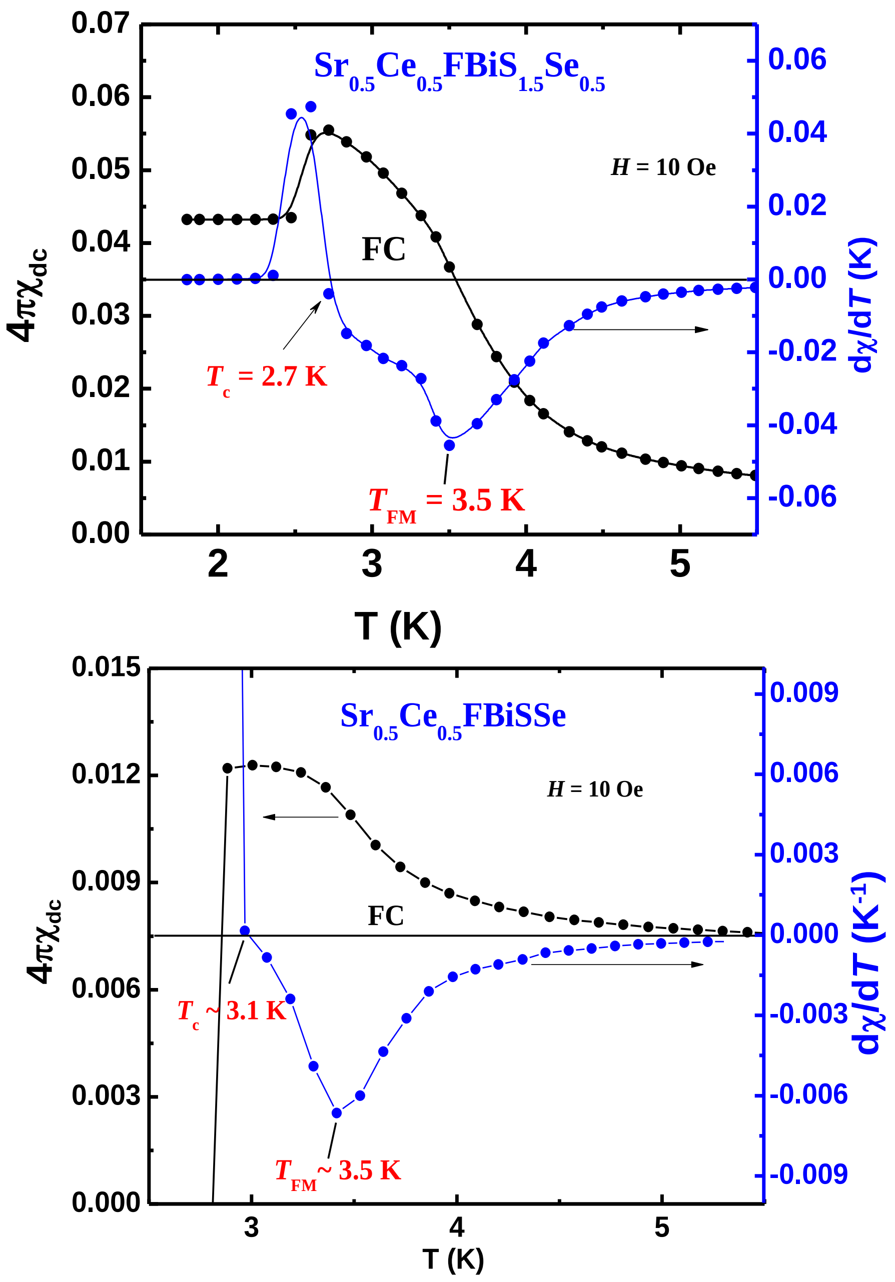

**Figure S3.** Variable temperature paramagnetic susceptibility (black) and its inverse (green) for $Sr_{0.5}Ce_{0.5}FBiS_{1.5}Se_{0.5}$ (a) and $Sr_{0.5}Ce_{0.5}FBiS_{1.0}Se_{1.0}$ (b). Red line is the Curie–Weiss fit.

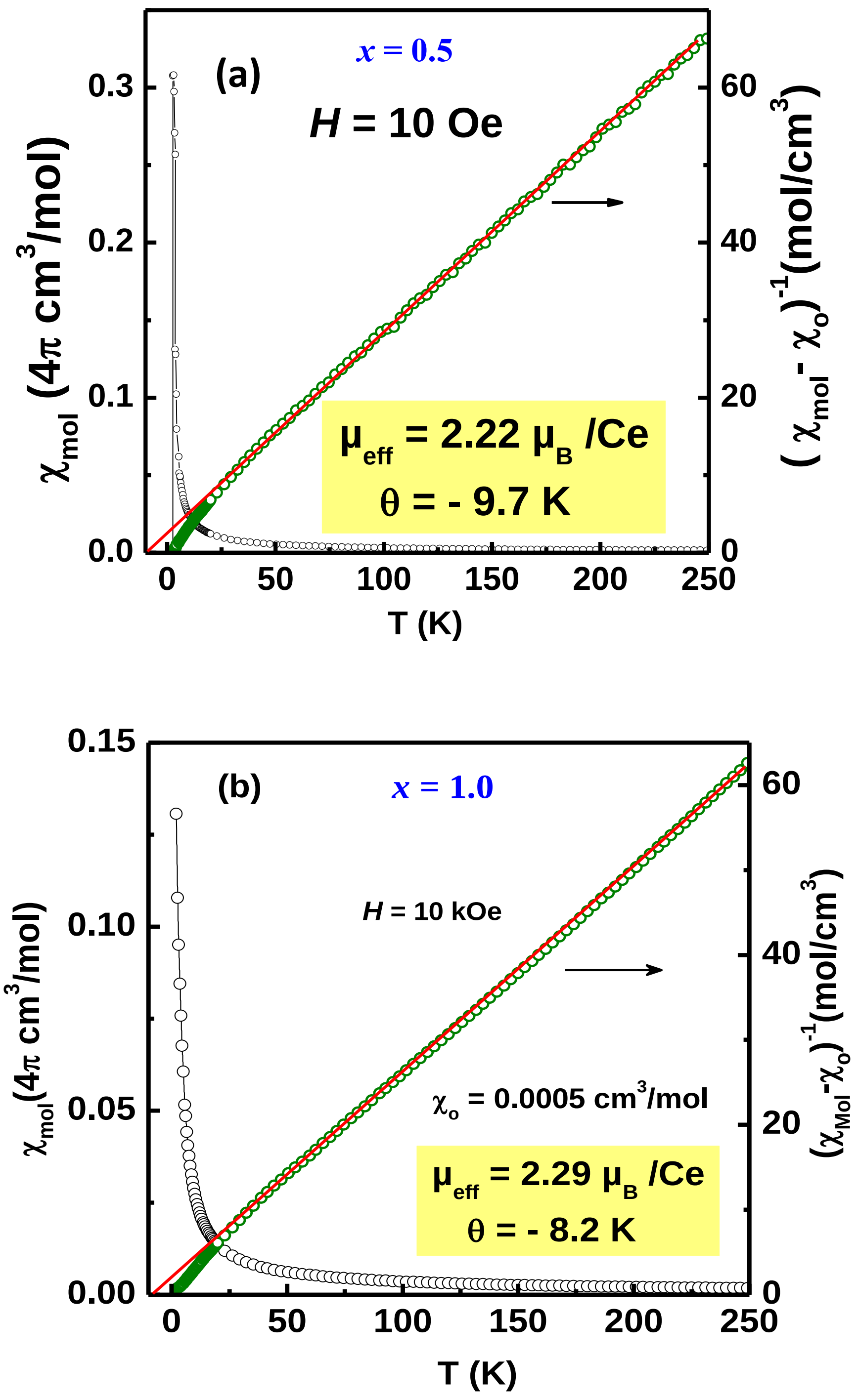

Supplement: Supplementary Information [file srep37527-s1.pdf]
